# Retrospective analysis of the clinical features of 172 patients with *BCR-ABL1*-negative chronic myeloproliferative neoplasms

**DOI:** 10.1186/s13039-020-0471-z

**Published:** 2020-02-17

**Authors:** Xiaolan Lin, Huifang Huang, Ping Chen

**Affiliations:** Fujian Institute of Hematology, Fujian Provincial Key Laboratory of Hematology, Fujian Medical University Union Hospital, 350000, Xinquan Rd, Fuzhou, Fujian China

**Keywords:** MPNs, Cytogenetics, Gene mutation, *JAK2-V617F*

## Abstract

**Background:**

To explore the clinical features of the patients with *BCR-ABL1*-negative chronic myeloproliferative neoplasms (MPNs) in our hospital and to reveal the unique features of *BCR-ABL1*-negative MPNs patients in our center.

**Methods:**

Retrospective analysis of routine karyotype analysis results, driver gene mutations and other related clinical parameters of 172 patients with newly diagnosed *BCR-ABL1*-negative MPNs who were admitted to our hospital between October 2013 and June 2018.

**Results:**

(1) The rate of karyotypic abnormalities were 25, 6.3 and 2.9% in primary myelofibrosis (PMF), polycythemia vera (PV) and essential thrombocythemia (ET) patients, respectively. (2) The mutation rate of *JAK2-V617F* was 62.5%, and that of the *CALR*, *MPL* and *EZH2* genes was 4.2% in PMF. The mutation rates of *JAK2-V617F* and *JAK2-12*exon were 91.3 and 1.3% in PV, respectively. The mutation rates of *JAK2-V617F* and CALR were 69.1 and 11.8% in ET, respectively. (3) Patients with *JAK2-V617F mutation* than with the wild-type gene were more often female in PMF (*P* = 0.027); had higher peripheral blood white blood cell (WBC) counts (*P* = 0.006), platelet (PLT) count (*P* = 0.001) and splenomegaly (*P* < 0.05) in PV; and had higher WBC (*P* = 0.001), hemoglobin concentrations (*P* = 0.001), lower PLT (*P* = 0.037), splenomegaly and endogenous coagulopathy (*P* < 0.05) in ET. (4) Among the PV and ET patients, those with thrombus were older than those in the nonthrombotic group.

**Conclusion:**

PMF patients have more chromosomal abnormalities than PV and ET patients, and the effect of driver mutations on the clinical features of patients with MPNs differs among the three subtypes.

## Background

Chronic myeloproliferative neoplasms (MPNs) consist of a group of clonal hematopoietic stem cell diseases characterized by sustained proliferation of myeloid or multilineage myeloid cells. Among MPNs, polycythemia vera (PV), essential thrombocythemia (ET), and primary myelofibrosis (PMF) are classified as *BCR-ABL1*-negative myeloproliferative neoplasms. Clinical manifestations include cytopenia of one or more lineages, thrombosis, myelofibrosis, varying degrees of extramedullary hematopoiesis and risk of leukemia conversion [1]. In recent years, with the advances of research in genetics and molecular biology, the pathogenesis, diagnosis, prognostic evaluation and treatment of such diseases have also progressed [2]. In the PMF patients, the international prognostic scoring system DIPSS-plus including cytogenetic indicators has been established, in which complex karyotype and karyotype abnormalities involving rearrangement of + 8, − 7/7q-, i(17q), − 5/5q-, 12p-, inv.(3) or 1lq23 have been assigned to the poor prognosis group [3]. However, whether cytogenetic abnormalities should be included in the risk stratification scheme of ET or PV is still controversial. In 2016, WHO included *JAK2*, CALR and *MPL* mutations in the main diagnostic criteria of MPN and recommended the detection of gene mutations such as *TET2, ASXL1, EZH2* and *SRSF2* for triple-negative MPNs [2]. In this article, we retrospectively analyzed the cytogenetics, distribution of driver genes and clinical parameters of 172 patients with newly diagnosed *BCR-ABL1*-negative MPNs in our hospital.

## Results

### General condition of 172 patients with *BCR-ABL1*-negative MPNs

Of the 172 patients, 101 were male and 71 were female; the male-to-female ratio was 1.4:1. The age of onset was 18 to78 years old, and the median age was 59 years. There were 24 patients with PMF, consisting of 17 males and 7 females. The male-to-female ratio was 2.42:1, the age ranged from 42 to 78 years old, the median age was 65.5 years, the median WBC was 12.26 × 10^9^/L (1.25–51.8 × 10^9^/L), the Hb level was 90.24 ± 34.98 g/L, and the median PLT 183 × 10^9^/L (1–806 × 10^9^/L). There were 80 patients with PV, 49 males and 31 females. The male-to-female ratio was 1.58:1, the age ranged from 21 to 78 years old, and the median age was 58 years old; the median WBC was 11.92 × 10^9^/L (4.34–50.25 × 10^9^/L), the. Hb level was 194.73 ± 20.66 g/L, and the median PLT 384 × 10^9^/L (93–1328 × 10^9^/L). There were 68 patients with ET, 35 males and 33 females. The male-to-female ratio was 1.06:1, the age ranged from 18 to 77 years old, the median age was 57 years old, and the median WBC was 11.31 × 10^9^/L (5.32–60.97 × 10^9^/L). The Hb level was 136.99 ± 26.74 g/L, and the median PLT median 878 × 10^9^/L (416–2243 × 10^9^/L).

### Cytogenetic analysis of 172 patients with *BCR-ABL1*-negative MPNs

The incidence of clonal chromosomal abnormalities in patients with PMF was 25% (6/24), that in patients with PV was 6.3% (5/80), and that in patients with ET was 2.9% (2/68). In addition, the total incidence rate of abnormalities in 172 patients was 7.6%. Thirteen cases of chromosomal abnormalities are shown in Table 1. The karyotype abnormality rate of patients with PMF was significantly higher than that of patients with PV and ET (*P* < 0.005), and the difference between PV and ET groups was not statistically significant (Table 3).

**Table 1:** The karyotypes of the 13 cases with abnormal chromosome

Disease	Karyotypes
PMF	46,XY,del(11)(q21)[20]
	46,XY,-20,+mar[11]
	44~46,XY,t(8;17)(q1?3;q11)[CP3]/46,XY[7]
	46,XY,t(8;9)(p22;p22)[5]
	der(15)
	46,XY,t(11;22)(q25;q11.2),add(18)(p11.3)[20]
PV	46,XY,add(6)(q25),del(9)(q13)[8]/46,XY,del(6)(p23)[2]/46,XY[10]
	46,XY,del(20)(q13.1q13.3)[10]
	46,XX,t(3;13)(q29;q12)[13]
	46,XX,t(6;8)(p22;q21)[3]
	46,XY,del(5)(q14q23)[13]
ET	46,XY,add(15)(q26)[11]/46,XY[9]
	46,XX,dic(1;1)(q44;p11)[20]

### The occurrence of gene mutation in 172 patients with *BCR-ABL1-* negative MPNs

In 172 patients, 135 cases *JAK2-V617F* mutations were detected; the total mutation rate was 78.5%. The detection rate of *JAK2-V617F* gene mutation in PMF patients was 62.5% (15/24); 1 patient was negative for mutation, 1 patient had *MPL* gene mutation, 1 patient had *CALR* mutation, and 1 patient had *EZH2* gene mutation; the overall mutation rate was 4.2%. The detection rate of *JAK2-V617F* gene mutation in PV patients was 91.3% (73/80). One of the 7 patients who was negative for mutations had a *JAK2-12*exon gene mutation with a mutation rate of 1.3%. The detection rate of the *JAK2-V617F* gene mutation in ET patients was 69.1% (47/68). Eight of the 21 patients with mutations had *CALR* mutation, and the mutation rate was 11.8% (8/68) (Table 2). The *JAK2-V617F* mutation rate in PV was significantly higher than that in PMF and ET (*P* < 0.01); the difference between the PMF and ET groups was not statistically significant (Table 3).

**Table 2:** Distribution of driven gene mutations (n(%))

Disease	JAK2-V617 mutation	Non-JAK2-V617 mutation				No driven gene mutations
		CALR	MPL	JAK2-12exon	EZH2	
PMF	15(62.5)	1(4.2)	1(4.2)	0	1(4.2)	6(25)
PV	73(91.3)	0	0	1(1.3)	0	6(7.5)
ET	47(69.1)	8(11.8)	0	0	0	13(19.1)

**Table 3:** Clinical features and outcome of three groups

Index	PMF (*n* = 24)	PV (*n* = 80)	ET (*n* = 68)	P			
				Whole group	PMF vs. PV	PMF vs. ET	PV vs. ET
Age(year)	65.5(42–78)	58(21–78)	57(18–77)	0.122			
sex				0.180			
male	17	49	35				
female	7	31	33				
abnormal chromosome	6	5	2	< 0.001^*^	0.003^*^	0.001^*^	0.686
JAK2 V617Fmutation	15	73	47	< 0.001^*^	0.007^*^	0.723	0.005^*^
Splenomegaly	24	58	23	< 0.001^*^	0.003^*^	< 0.001^*^	< 0.001^*^
Thrombosis	2	13	12	0.550	0.511	0.342	0.821
hemorrhage	1	5	1	0.341	0.578	0.456	0.219
Leukaemia	1	0	0	0.045	0.231	0.261	/
Bone fibrosis	/	2	3	0.661			0.661
High LDH	24	56	39	0.001^*^	0.002^*^	< 0.001^*^	0.110

### Concomitant disease and disease outcome

There were 27 cases of current or previous thrombosis in 172 patients, including 22 cases of cerebral infarction, 1 case of cerebral infarction with iliac vein thrombosis, 2 cases of myocardial infarction, 1 case of splenic vein thrombosis, and 1 case of pulmonary artery and calf vein thrombosis; the thrombosis rates of PMF, PV and ET were 8.3, 16.3, and 17.6%, respectively. Additionally, there were 7 cases of bleeding, including 3 cases of gingival bleeding, 3 cases of cerebral hemorrhage, and 1 case of gastrointestinal bleeding. No significant differences were detected with regards to thrombosis, bleeding events, leukemia, and progression of bone marrow fibrosis among the three groups. The level of spleen enlargement was higher than that in PV, which was greater than that in ET. The difference among these groups was statistically significant (*P* < 0.001). The level of LDH in PMF was greater than that in PV and ET (*P* ≤ 0.002); the difference between PV and ET was not statistically significant. (Table 3).

### Comparison of the clinical parameters of MPN patients with mutant and wild-type *JAK2-V617F*

The clinical parameters included gender, age, peripheral blood WBC count, Hb concentration, PLT count, splenomegaly, thrombotic events, LDH, coagulation function and chromosomal abnormalities. The results are shown in Table 4. Compared with patients with wild-type genes, more patients with PMFs were female (*P* = 0.027). PV patients carrying a *JAK2 V617F* mutation had higher peripheral blood WBC counts (*P* = 0.006) and PLT counts (*P* = 0.001) and were more prone to splenomegaly (*P* < 0.05). ET patients carrying a *JAK2 V617F* mutation had higher peripheral blood WBC counts (*P* = 0.001), higher Hb concentrations (*P* = 0.001), and lower PLT counts (*P* = 0.037) and were more prone to splenomegaly and abnormal endogenous coagulation (*P* < 0.05).

**Table 4:** Comparison of clinical parameters between JAK2-V617F mutant and wild type in three groups of patients

Index	PMF		P	PV		P	ET		P
	M^※^(*n* = 15)	W^*^(*n* = 9)		M(*n* = 73)	W(*n* = 7)		M(*n* = 47)	W(*n* = 21)	
Media age(y)	69(42–78)	58(47–70)	0.122	58(22–78)	49(21–74)	0.821	59(28–81)	58(18–85)	0.532
Sex									
Male	8	9	0.027^*^	42	6	0.236	23	12	0.605
Female	7	0		30	1		24	9	
WBC(10^9^/L)	7.5(2.7–23.1)	20.7(1.3–51.8)	0.497	12.6(4.3–50.3)	6.8(4.4–12.7)	0.006^*^	12.5(6–61)	8(5.3–26)	0.001^*^
Hb(g/L)	90.7 ± 29.1	87.1 ± 46.0	0.303	193.7 ± 19.8	207.4 ± 29.6	0.089	144.1 ± 24.3	121 ± 25.6	0.001^*^
PLT(10^9^/L)	147(62–617)	192(1–806)	0.61	404(93–1328)	217(93–305)	0.001^*^	813.5(538–2240)	985(416–2243)	0.037^*^
Splenomegaly	15	9	/	55	3	0.045^*^	20	3	0.023^*^
Thrombosis	1	1	0.62	12	1	0.859	9	3	0.742
Bone fibrosis				2	0	0.657	2	1	0.676
APTT extension	9	7	0.332	58	5	0.636	28	4	0.002^*^
PT extension	4	2	0.601	25	3	0.691	4	0	0.168
Abnormal FIB	10	4	0.262	14	3	0.161	10	4	0.273
High D-Di	9	6	0.547	20	2	0.625	15	9	0.383
High LDH	15	9	/	50	6	0.668	25	14	0.299
Karyotype analysis									
Normal	12	6	0.499	68	7	0.685	45	21	0.475
Abnormal	4	3		4	0		2	0	

※M = mutation

*W=Wild

### Comparison of the laboratory and clinical indicators of the thrombotic and nonthrombotic groups

Color Doppler Ultrasound or angiography were used to assess the clinical data patient to verify the thrombotic condition. Compared with the non-thrombotic group, the thrombotic group of PV and ET patients had higher age characteristics (*P* < 0.05). However, there were no statistically significant differences between the other observed clinical indicators or in the indicators between the two groups of PMF patients.

## Discussion

MPN is a group of heterogeneous diseases originating from hematopoietic stem cells. It is also a collective term for a group of neoplastic diseases characterized by clonal proliferation of one or more lineages of relatively mature bone marrow cells. No reliable epidemiological investigation in China has been reported. In our hospital, the age of incidence of *BCR-ABL1*-negative MPNs is 18–78 years old. The incidence rate of middle-aged and elderly people is the highest, the median age of onset is 59 years old, and the ratio of male to female is 1.4:1.

The total incidence of abnormal MPN karyotypes in this study was 7.6%, including 25% for PMF, 6.3% for PV, and 2.9% for ET; the PMF karyotype abnormality was the highest. However, in foreign research reports, the proportion of PMF karyotype abnormalities is 33–55% [4–7]. However, the data at our center were roughly consistent with the incidence of PMF chromosomal abnormalities reported by other centers in China [8]. The most common of the karyotype abnormalities was trisomy 8 (28%), followed by the complex anomaly (19%) and 20q- (15%). Six chromosomal abnormalities were observed in 24 PMF patients in our hospital, and none of them showed + 8. The abnormalities seen were − 20, +mar, del(11q), der(15), add(18p) and three equilibrium translocations: t(8;17), t(8;9), and t(11;22). One of the patients died of pulmonary infection (karyotype 46,XY,-20,+mar[11]) 22 days after the initial diagnosis. Another patient converted to acute monocytic leukemia at the initial diagnosis at 53 months (karyotype 46,XY,del(11)(q21) [20]). Many foreign studies have shown [9, 10] that the incidence of karyotype abnormalities in the initial diagnosis of PV was 15 to 25%; common abnormalities included del(20q),+ 8, + 9, in addition to dup(1q), del(13q), and del(5q). The rate of chromosomal abnormality in PV patients in this study was lower than that reported in the foreign literature, but it was consistent with Limin Duan et al. reports [11]. The abnormalities included del(20q), del(5q), add(6q), del(6p), and del(9q) and two equilibrium translocations t(3;13) and t(6;8). One patient developed bone marrow fibrosis 72 months after diagnosis (karyotype 46,XY,del(20)(q13.1q13.3)[10]). The rate of chromosomal abnormality of ET patients was the lowest in this study, consistent with Lan HF et al. reports [12]. Only 1 case with add(15q) and dic(1;1) was abnormal. In summary, the rate of karyotype abnormality in patients with MPNs in this study is different from that in other reports, may be related to the genetic background of different ethnic groups. It is also possible that karyotype analysis consisted mostly of normal cells rather than tumor cells due to the susceptibility to blood thinning in patients with MPN.

In this study, the *JAK2-V617F* mutation had the highest detection rate in PV (91.3%), relative to ET (69.1%) and PMF (62.5%), which was consistent with the literature [13–16], followed by the *CALR* gene mutation. The mutation rates in PMF and ET were 4.2 and 11.8%, respectively, but they were not detected in PV. The detection rate reported by Tefferi [17] and Haslam [18] was significantly lower than that detected by Rotunno [19], which may be related to the small number of specimens, the error rate of the results, the sensitivity of the experimental methods, and the lack of screenings for three driver genes in some patients. The *MPL* gene and *JAK2 -12*exon mutations were rare: only one *MPL* mutation was found in PMF (4.2%), and one *JAK2 -12*exon mutation (4.2%) was found in PV, which was consistent with foreign reports [20, 21]. Chromatin-modifying genes (*ASXL1* and *EZH2*) were associated with transformed acute leukemia, and Delic et al. [22] used second-generation gene sequencing to determine that *ASXL1* and *EZH2* mutations were frequently mutated in PMF patients but rarely mutated in ET and PV patients. Thus it may be the reason that higher rate of transformation of AML in PMF patients than ET and PV patients. In this study, one case of *EZH2* mutation was also detected in patients with PMF. This patient has not been discharged after multiple treatments but has not converted to leukemia.

Tefferi and others [23, 24] found that patients with *JAK2-V617F* mutation-positive PMF had higher WBC counts and lower Plt counts in peripheral blood, but in this study, the *JAK2-V617F* mutant in PMF showed only gender differences compared with wild-type genes. This result may be related to the fact that the bone marrow of PMF patients often failed to be cultured due to a dry tap, resulting in a small quantity of specimens. Patients with PV with *JAK2-V617F* mutation in this study were associated with higher WBC and Plt counts and larger spleens, similar to HE Zhipeng et al. reports [25]. In contrast, foreign reports of *JAK2-V617F* mutations in PV patients were associated with higher Hb levels and lower Plt counts [21], which may be related to ethnic differences. ET with *JAK2-V617F* mutation in this study was associated with higher WBC and Hb and lower Plt, consistent with previous reports [23, 24]. It was also found to be associated with a larger spleen and was more susceptible to endogenous coagulopathy than in a negative patient.

Bleeding and thrombosis are common complications of MPN and have an important impact on morbidity and mortality. This study showed that the incidence of thrombosis in PMF, PV, and ET was 8.3, 16.3, and 17.6%, respectively, which was consistent with reports by Falanga [26]. Some scholars have analyzed the clinical features and prognosis of patients with PMF and found that patients with *JAK2-V617F* mutation-positive PMF have an increased incidence of thrombosis. Those with homozygous mutations were more likely to develop leukemia [27]. The study of PV patients found that the age of onset in the *JAK2-V617F*-positive group was higher than that in the negative group, which was clinically more prone to complications of myelofibrosis, hemorrhage and thrombosis [28]. De Grand et al. [29] found that *JAK2-V617F* mutations in PV patients could increase the risk of thrombosis by activating Lu/BCAM-mediated red blood cell adhesion aggregation. Lu et al. [30] also found that JAK2-dependent signaling pathways played an important role in platelet activation in PV patients and increase the risk of thrombosis. No significant differences in the thrombosis and hemorrhagic events were observed between the three groups of diseases and the mutant and wild-type *JAK2-V617F* in this study, which may be related to the number of cases or ethnic differences. In PV and ET patients, the thrombotic group was older than the nonthrombotic group. Older patients had slower blood flow, increased viscosity, arteriosclerosis, and severe damage to vascular endothelial cells. At the same time, the incidence of thrombotic diseases such as diabetes and hypertension was high, which may lead to thrombosis. Therefore, anticoagulation intervention in elderly patients can help reduce the occurrence of thrombosis. Dunlap, J. et al. [31] reported that the frequency of cytogenetic abnormality in the *JAK2*+ group was higher than that in the *JAK2*-group (23/45 (51%) vs 14/52 (27%), respectively). In this study, considering the chromosomal abnormalities of the three groups, no statistically significant difference was found in the rates in the *JAK2-V617F* mutation group and the wild-type group, which may be related to the small number of enrolled cases.

LDH can reflect the rate of cell replication and apoptosis. Many studies have found that high serum LDH is found in PMF and progressive MF (post-PV and post-ET) and is included in the secondary diagnostic criteria [32]. Through multivariate analysis studies, Mohamad Cherry et al. [33] found that age and high levels of LDH were negatively correlated with overall survival in patients with ET and PV. In this study, the elevated level of LDH in PMF was significantly greater than that in PV and ET, but the difference between PV and ET was not statistically significant. This also indicated that the tumor burden of PMF was higher than that of PV and ET, while there was no significant difference in the elevated levels of LDH between mutant and wild-type *JAK2-V617F* in the three groups.

In summary, MPN is a heterogeneous group of diseases. This study describes the cytogenetics, gene mutations, and clinical features of each disease. With the advancement of gene detection technology, different types of *CALR* mutations, *MPL* gene mutations and triple-negative mutations may have further implications.

## Conclusion

We provided an important information of cytogenetics,gene mutation and clinical features of MPNs in our region. We found that PMF patients have more chromosomal abnormalities than PV and ET patients, and the types of driver mutations are closely related with the clinical features in *BCR-ABL1*-negative MPN patients.

## Materials and methods

### Study design

The study retrospectively analyzed 172 patients with *BCR-ABL1*-negative MPNs who were newly diagnosed in our hospital between June 2013 and June 2018. The patients’ diagnoses were established according to the 2016 WHO diagnostic criteria [2].

### Observational index

Clinical data of the patients with PMF, PV and ET included age of onset, gender, white blood cell (WBC) count during blood routine examination on admission, hemoglobin (Hb) and platelet (PLT) counts, coagulation (PT, APTT, FIB, D-Di) and LDH, karyotype analysis and mutations such as *JAK2-V617F*, concomitant disease (splenomegaly, thrombosis) and disease outcome.

### Bone marrow karyotype analysis

Five to six milliliters of bone marrow was collected from patients and anticoagulated with heparin. After counting, the chromosomes were prepared by direct and short-term culture methods with (1 ~ 2) × 10^6^/mL, and karyotype analysis was performed by R Banding. Karyotype abnormalities were identified and described in accordance with the International System for Human Cytogenetics (ISCN, 2013) [34].

### Gene detection

PCR or Sanger sequencing was used to detect mutations in the *JAK2*, *CALR*, and *MPL* genes*.* Patients who harbored no mutation in these three genes were further tested for the presence of *TET2, ASXL1, EZH2* and *SRSF2* mutations. A ratio of the copy number of the target gene to the copy number of the reference gene × 100% greater than 1% was taken as a positive result [35]. A volume of 3 mL of the patient’s bone marrow anticoagulated with EDTA was subjected to DNA extraction (Sangon). The concentration and purity of the DNA product were measured by ultraviolet spectrophotometry (Thermo NanoDrop). The PCR primers were added to carry out the PCR. The PCR products were electrophoretically isolated, purified, and subjected to bidirectional gene sequencing using an ABI 3730 sequencer. The sequencing results were compared with GenBank data to identify the type of gene mutation.

### Statistical analysis

Statistical analyses were performed by using SPSS 17.0 statistical software. The mean values of the measured data were compared by analysis of variance (ANOVA) or the Kruskal-Wallis test. The count data were compared by the χ^2^ test. Differences with *P* < 0.05 were considered statistically significant.

## Data Availability

All data generated or analyzed during this study are included in this published article and its additional files.

## Abbreviations

AML: Acute myeloid leukemia
ASXL1: Additional sex combs like 1
CALR: Calreticulin
DIPSS-plus: Dynamic International Prognostic Scoring System-plus
ET: Essential thrombocythemia
EZH2: Enhancer of zeste homolog 2
ISCN: International System for Human Cytogenetics
JAK2: Janus kinase 2
MPL: Myeloproliferative leukemia virus
MPN: Myeloproliferative neoplasms
PCR: Polymerase chain reaction
PMF: Primary myelofibrosis
PV: Polycythemia vera
SRSF2: Serine and arginine rich splicing factor 2
TET2: Ten-eleven translocation oncogene family member 2
